# Women's Experiences of Preeclampsia: Australian Action on Preeclampsia Survey of Women and Their Confidants

**DOI:** 10.1155/2011/375653

**Published:** 2011-03-23

**Authors:** C. East, K. Conway, W. Pollock, N. Frawley, S. Brennecke

**Affiliations:** ^1^Department of Obstetrics & Gynaecology, The University of Melbourne and Department of Perinatal Medicine, Royal Women's Hospital, 20 Flemington Road, Parkville, VIC 3052, Australia; ^2^Australian Action on Pre-Eclampsia, P.O. Box 29, Carlton South, VIC 3053, Australia; ^3^Division of Nursing & Midwifery, La Trobe University/Mercy Hospital for Women, 163 Studley Road, Heidelberg, VIC 3084, Australia; ^4^Department of Perinatal Medicine, Royal Women's Hospital, 20 Flemington Road, Parkville, VIC 3052, Australia

## Abstract

*Introduction*. The experience of normal pregnancy is often disrupted for women with preeclampsia (PE). *Materials and Methods*. Postal survey of the 112 members of the consumer group, Australian Action on Pre-Eclampsia (AAPEC). *Results*. Surveys were returned by 68 women (61% response rate) and from 64 (57%) partners, close relatives or friends. Respondents reported experiencing pre-eclampsia (*n* = 53), eclampsia (*n* = 5), and/or Hemolysis, Elevated Liver enzymes, and Low Platelets (HELLP syndrome) (*n* = 26). Many women had no knowledge of PE prior to diagnosis (77%) and, once diagnosed, did not appreciate how serious or life threatening it was (50%). Women wanted access to information about PE. Their experience contributed substantial anxiety towards future pregnancies. Partners/friends/relatives expressed fear for the woman and/or her baby and had no prior understanding of PE. *Conclusions*. The PE experience had a substantial effect on women, their confidants, and their babies and affected their approach to future pregnancies. Access to information about PE was viewed as very important.

## 1. Introduction

Preeclampsia (PE) is a pregnancy specific, heterogeneous, multisystem disorder, which has the classic clinical features of pregnancy-induced hypertension and proteinuria and may lead to eclampsia (E) [1–3]. The presence of pregnancy-induced Hemolysis, Elevated Liver enzymes, and Low Platelets (HELLP syndrome) may also be classified as a form of preeclampsia [4]. Preeclampsia, eclampsia, and HELLP syndrome are a significant cause of maternal and perinatal morbidity, mortality, and iatrogenic premature delivery [5], with long-term health effects for both mother and child [6, 7]. Considerable research efforts have resulted in improved understanding of the genetic basis of preeclampsia [8], exploration of numerous possible predictors of the disorder [9], and resulted in the use or nonuse of diverse treatments [10–18]. Of these possible treatments, antihypertensive medications and magnesium sulphate are widely used in developed countries and in some developing countries [19]. Delivery of the baby, placenta, and membranes, indicated for maternal or fetal reasons, remains the only method for resolving preeclampsia, although it does not immediately remove all risks of mortality and morbidity, particularly in the early postpartum period. Thus, there is considerable understanding of the genetic, pathophysiological, and clinical manifestations of PE, effective treatments, and long-term physical effects. 

There is, however, a paucity of published research on women's perceptions of their experiences of PE, which may impact on their lives well beyond delivery and the puerperium. Research in this area has largely focused on the relationship of PE to posttraumatic stress disorder (PTSD). Poel et al. [20] surveyed a single tertiary hospital's inpatients with PE and found that 18% of patients with PE required a referral to a psychologist for dysfunctional coping, and this included 3% who were diagnosed with PTSD. Van Pampus et al. [21] detailed three women's experiences with severe PE and highlighted the heavy burden patients with PE may have—not only a serious illness in the mother, an unexpected and potentially medicalized delivery, but also often a premature baby, all of which are hypothesised to have a trend toward association with PTSD. 

The experiences of eight women with severe early onset preeclampsia were explored in a qualitative study [22]. Common themes in these families included a feeling that something was not right in the pregnancy, feeling a loss of control or “thrownness,” fear for the baby's prematurity, loss, guilt, and the challenge of the illness and recovery itself. It is interesting that even though PE is actively screened for at each antenatal visit with at least a measurement of the woman's blood pressure, the diagnosis still came as a complete shock to the majority of women studied.

Cowan [22] acknowledged that it is the atypical and variable nature of PE that contributes to patients having a frustrating experience with their diagnosis. Often during the initial phase when PE is evolving, women may have vague symptoms, and there may be many visits before the diagnosis is actually determined. Women who have atypical presentations in particular, such as the absence of proteinuria in HELLP syndrome, may have a delayed diagnosis and further frustration with their medical care. Antenatal hospitalisation for conditions such as preeclampsia has also been suggested to lead to maternal identity change which may have ongoing psychological consequences [23].

The consumer group, Australian Action on Pre-Eclampsia (AAPEC) [24], is a voluntary organisation set up to provide support and information to families who have experienced PE/E. It also aims to educate, inform, and advise the public and health professionals about the PE/E.

## 2. Materials and Methods

The AAPEC executive, including the then President (K. Conway), Medical Advisor (S. Brennecke), Midwifery Advisor (C. East) and several active members, formed a working party to develop a survey to gain a general overview of how PE was experienced by their membership and help AAPEC better meet the needs of current and future members. Further input from a critical care obstetric viewpoint (W. Pollock) was also obtained.

The key themes from the work by Cowan [22] were supplemented with themes identified by the working party, to develop relevant questions. Considerable attention was given to the inclusion of questions about the death of a member's baby, with wording developed and piloted by members to ensure appropriateness. Women whose baby had died during or following a pregnancy complicated by PE were invited to complete further questions as they chose. Questions about mothering, for example, were completed by some who lost their baby and also by those who may have subsequently had a baby. All questions were piloted among a small group of members, and their feedback was incorporated into the final survey. 

The questions sought information about the woman's PE/E, history, timing of diagnosis of PE/E and outcomes, including level of clinical care for the mother and baby (for example, admitted to an intensive care unit). We sought to quantify women's level of knowledge, prior to diagnosis, of their potential to develop PE/E and the implications that such a condition could have for themselves and their babies, as well as their level of acceptance and understanding of the diagnosis once made. Several questions explored aspects of feeling in control of their lives and destiny, whether women considered that clinical staff believed them when they presented with symptoms and women's understanding of how sick they were. Further thematic exploration involved the implications of premature and/or emergency delivery and how women coped with separation from their baby, due to their own or their babies' health needs. We also sought information about women's sense of self-worth, mothering ability, and recovery from the illness, how they perceived their experience had affected or might influence their decisions and care for future pregnancies, as well as what information they would have found beneficial before and following the development of PE/E. Response options included “strongly agree,” “agree,” “disagree,” “strongly disagree,” or “undecided”; “none,” “a little,” “very little,” “not at all,” “undecided,” as appropriate. Results were summarized in order to simplify them and highlight the more meaningful findings, for example, “strongly agree” and “agree,” or “disagree” plus “strongly disagree,” as appropriate for each question.

A section of the survey was devoted to exploring the perceptions of one of each woman's social contacts, including, as selected by each woman, her partner, close family member or friend. We sought to elicit how these confidants felt about the likelihood that PE/E could develop, specifically for someone they know, fear about outcomes, including death of the woman and/or her baby and feeling that they may or may not have known what to do to help.

The surveys, with an accompanying information sheet and reply paid envelope for return, were posted to each of the 112 AAPEC members. A follow-up survey was posted approximately six weeks later, to enhance the response rate. No identifying data were noted on the survey and a unique identifier was allocated to all returned surveys. Consent was implied by return of the survey, and the project was approved by the Royal Women's Hospital Human Research Ethics Committee.

Data were entered onto a database and analysed using descriptive statistics (Microsoft Access, Microsoft Excel, Microsoft Corp, Redmond, WA; SPSS version 16.0 software, SPPS Inc, Chicago, IL, USA).

## 3. Results

Surveys were returned by 68 women (61% response rate) and from 64 partners, close relatives, or friends (57% response rate). Characteristics, outcomes, and responses are provided in Tables 1 and 2. Respondents reported experiencing preeclampsia (*n* = 53), eclampsia (*n* = 5), and/or HELLP syndrome (*n* = 26) within a median of four years prior to the survey (range 1 to 47 years). Many felt that “something was not quite right” and, once diagnosed, that this could not be happening to them. Although only 19% did not believe the initial diagnosis, 51% of respondents thought that PE was not serious or life threatening (Table 2). Fifty eight women had given birth prematurely (between 30 weeks and 36 weeks 6 days, *n* = 31; before 30 weeks, *n* = 27), and eighteen babies had died (Table 1). Most women whose babies had died noted that they felt well supported by hospital staff (*n* = 14). Women felt that their experience with PE had a substantial effect on their anxiety towards future pregnancies and the level of medical care for subsequent pregnancies.

Partners/friends/relatives expressed fear that the woman and/or her baby could have died, that they had no prior awareness of PE, and that they did not know what to do to help (Table 3).

## 4. Discussion

The PE experience had a substantial effect on women, their confidants, and their babies in the index pregnancy. The diagnosis was a shock to many respondents, and there were elements of denial of the severity of the condition until after the event. Prior to the diagnosis of PE, many women may have never experienced a significant illness and had been expecting a routine, normal pregnancy. Many of the experiences recounted by the women were emotionally intense and can be interpreted in the context of anxiety, depression, and posttraumatic stress disorder [21, 25]. Several of the known risk factors for PTDS were elicited, including feelings of loss of control over their situation and obstetric procedures, commonly induction or caesarean section at an earlier time point than expected had the pregnancy been normal and, for some, extremely premature [21, 25]. For other women, the emotional intensity of the experience may not have equated with a diagnosis of PTSD, but our findings indicated that it was no less important for the individual woman and her support network. 

The experiences of women whose babies are cared for in the neonatal intensive care nursery, including those born preterm, have some similarities, particularly issues of bonding when the infant is too small or too sick to be breastfed or handled. The provision of emotional support to the mother is often rated very well in the intensive care setting [26] and forms an important component of processing the feelings engendered by the experience [27]. Women in our survey reported that their experience with bonding was not only limited by the baby's health but also their own, with some women being in intensive care settings themselves. Further exploration of women's experiences using qualitative methodologies may provide additional insights to identify and promote interventions to assist with this.

The PE experience affected how women approached future pregnancies, as well as the level of medical care and choice of hospital for the subsequent pregnancy and birth. This has implications for the provision of antenatal and birthing services for the small percentage that may experience PE in a subsequent pregnancy [28]. Australia's population is largely clustered around the major cities, with concurrent centralisation of specialty healthcare facilities in these cities. Women requiring more intense surveillance throughout pregnancy who reside in regional centres may have been transferred to tertiary facilities in their index PE pregnancy, including the baby requiring neonatal intensive care in a tertiary setting. These women may need to relocate to be near a specialty healthcare facility for at least some of their subsequent pregnancy including possible induction of labour [26]. Such a move has major social and financial implications for the woman and her family—removing the woman from her social context at a time when her emotional needs are as important as her physical needs, and those of her baby only serve to perpetuate the cycle of morbidity and the potential for nonresolution of posttraumatic stress disorder if that is a feature for her. Studies of paternal experiences supporting their partner during pregnancy and childbirth have identified themes of feeling helpless, lacking knowledge, and potentially altering their relationship with their partner [29]. The experiences reported by the respondent's partner, close friend, or family member mimic those of the social networks of critical care patients, including the need to work through the shock of receiving the news, uncertainty surrounding the potential outcome for mother and baby, provision of adequate information, and involvement in decision making and moving on [30, 31].

Access to information about PE was viewed as very important. The origins of AAPEC [24] lie in women's perceived need to access information at a time when internet-based sites and hard copy publications were less prolific than they are now (personal communication, 13 October 2010). Although numerous sites can now be accessed with a simple online search, the quality and readability of information need to be appropriate for women with varying levels of health literacy [32]. 

This survey examined the experiences of PE within the AAPEC membership, with a reasonable response rate of 61%. Women who seek membership of such a consumer organisation and who respond to this type of survey may not be representative of all women who experience PE, and more research is required to examine the impact of PE on women and their confidants in the broader maternity services sector.

## 5. Conclusion

The responses to this survey from women and their partners, close relatives, or friends indicated that the experience of PE was viewed as very important to all and traumatic to many. The provision of information and support was valued prior to and at the time of diagnosis and needed to be revisited during ongoing care. Referral to consumer groups such as AAPEC [24] may be beneficial. Further research may consider the experiences of women and their confidants closer to the time of the PE experience.

## Figures and Tables

**Table 1:** Characteristics and outcomes of respondents.

	n*	(%)
Condition: one or more of		
Preeclampsia	53	(77.9)
Eclampsia	5	(7.4)
HELLP	26	(38.2)

Multiple conditions (included in the above)		
Preeclampsia and HELLP	13	(19.1%)
Preeclampsia and eclampsia	1	(1.5)
Eclampsia and HELLP	1	(1.5)
Preeclampsia, eclampsia, and HELLP	2	(2.9)

PE** experience		
One pregnancy	55	(80.9)
Two or more pregnancies	13	(19.1)

Highest level of care received		
Normal labour and postnatal care	5	(7.4)
Labour ward with additional care	24	(35.3)
Adult intensive care unit	39	(57.4)

Timing of delivery		
After 37 weeks	10	(14.7)
Between 30 weeks and 36 weeks 6 days	31	(45.6)
Before 30 weeks	27	(39.7)

Delivery earlier than planned		
No	4	(5.9)
Concern for maternal welfare	23	(33.8)
Concern for fetal welfare/fetal death	7	(10.3)
Concern for both mother and fetus	33	(48.5)

Perinatal/infant death		
Stillborn	9	(13.2)
Death within one week	4	(5.9)
Death one week to six weeks	3	(4.4)
Death six weeks to six months	1	(1.5)
Death after six months	1	(1.5)

*Not all answers completed by respondents.

**Hereafter, PE used to capture all of Preeclampsia, eclampsia, and HELLP.

**Table 2:** Women's reported perceptions of their PE* experience.

	*n*	%
Life perceptions and knowledge prior to diagnosis of preeclampsia		
No knowledge of PE	32	77.4%
Felt in control of own destiny	29	42.6%
Felt unwell	35	51.5%
Felt that something was not quite right	42	61.8%

Following diagnosis with PE		
Thought it could not happen to her	41	60.3%
Did not believe the doctor or midwife	13	19.1%
Thought it was not serious or life threatening	34	50.0%
Was frightened	49	72.1%
Felt a sense of letting self down by becoming sick with PE	43	63.2%

As the PE continued or became more severe		
Felt had lost control of own destiny	56	82.4%
Felt no longer “owned” the things that shaped own life	57	83.8%
Did not believe the doctors/midwives about being unwell	16	23.5%
Felt that doctors/midwives did not believe me that I was unwell	13	19.1%
Felt that no-one around had been through same experiences	50	73.5%

How the PE affected early experiences with the baby/babies		
It was a shock to know might give birth early	53	77.9%
Was frightened of how baby would manage if born early	52	76.5%
Was more worried about the baby than about self	55	84.6%
Felt that baby might die	41	60.3%

Feelings that ability to bond with baby were limited, because		
As a mother, was too sick	48	70.6%
Baby was too sick	39	57.4%
Baby and mother cared for in different parts of hospital	47	69.1%
Difficult to establish breastfeeding	35	51.5%

In the weeks, months, or years since the PE experience		
Had professional counseling to talk about the experience	25	36.8%
Felt the need to obtain more information about PE	66	97.1%
Found it was easy to obtain the information about PE	35	51.5%
Have fully recovered from the PE	50	73.5%
Have felt needed extra healthcare compared with women whose pregnancies were normal	55	82.1%
Found friends and/or family were very supportive and helpful	58	85.3%
Found talking or writing about PE experience was helpful	53	77.9%
Have had very little confidence in mothering ability	16	23.5%
Have had a strong sense of self-worth	44	64.7%
Believe have been a very successful mother	51	75.0%
Consider that baby has needed extra healthcare	26	38.2%
Have become aware that there may be a genetic link for PE, for example, own mother had PE	50	73.5%
Have been concerned about risk of PE if daughter/sister is pregnant	52	76.5%

How women considered their experience of PE affected later pregnancies		
Felt increased anxiety towards future pregnancies	64	94.1%
PE experience influenced interval to next pregnancy	37	54.4%
Enjoyment of the pregnancy	42	61.8%
Choice of primary caregiver (e.g., Midwife, General Practitioner, Specialist Obstetrician, Hospital Clinic)	49	72.1%
Choice of hospital	40	70.6%
Level of medical care during pregnancy	48	70.6%
How baby was born (e.g., by Cesarean Section)	33	48.5%

*“PE” used to capture all of preeclampsia, eclampsia, and HELLP.

**Table 3:** The PE experience from the perspective of the women's partners, family member, or close friends.

	*n*	%
Felt scared of losing the partner/relative/friend	49	76.6%
Felt scared that the partner/relative/friend may lose the baby	45	70.3%
Felt the need to advocate for the partner/relative/friend and baby	39	60.9%
Thought that most women have babies at 40 weeks with many problems	44	68.8%
Never expected the pregnancy to end like this	46	100.0%
Had never heard of preeclampsia before this	48	75.0%
Had heard a little about problems in pregnancy	51	79.7%
Never felt that pregnancy problems would happen to someone they knew	41	64.1%
Could see this coming because noticed the partner/relative/friend was unwell leading up to this	16	25.0%
Did not know what to do to help	46	71.9%
Felt was able to support partner/relative/friend as planned	40	58.8%

## References

[1] Lowe SA, Brown MA, Dekker GA (2009). Guidelines for the management of hypertensive disorders of pregnancy 2008. *Australian and New Zealand Journal of Obstetrics and Gynaecology*.

[2] Duley L (2008). Pre-eclampsia, eclampsia, andhypertension. *Clinical Evidence*.

[3] Duley L (2009). The global impact of pre-eclampsia and eclampsia. *Seminars in Perinatology*.

[4] Habli M, Eftekhari N, Wiebracht E (2009). Long-term maternal and subsequent pregnancy outcomes 5 years after hemolysis, elevated liver enzymes, and low platelets (HELLP) syndrome. *American Journal of Obstetrics and Gynecology*.

[5] Nelson-Piercy C (2006). *Handbook of Obstetric Medicine*.

[6] Anderson CM (2007). Preeclampsia: exposing future cardiovascular risk in mothers and their children. *Journal of Obstetric, Gynecologic, and Neonatal Nursing*.

[7] Wu CS, Nohr EA, Bech BH, Vestergaard M, Catov JM, Olsen J (2009). Health of children born to mothers who had preeclampsia: a population-based cohort study. *American Journal of Obstetrics and Gynecology*.

[8] Johnson MP, Roten LT, Dyer TD (2009). The *ERAP2* gene is associated with preeclampsia in Australian and Norwegian populations. *Human Genetics*.

[9] Meads CA, Cnossen JS, Meher S (2008). Methods of prediction and prevention of pre-eclampsia: systematic reviews of accuracy and effectiveness literature with economic modelling. *Health Technology Assessment*.

[10] Duley L, Henderson-Smart D (2000). Magnesium sulphate versus phenytoin for eclampsia. *Cochrane Database of Systematic Reviews*.

[11] Duley L, Henderson-Smart D (2000). Magnesium sulphate versus diazepam for eclampsia. *Cochrane Database of Systematic Reviews*.

[12] Duley L, Henderson-Smart DJ, Meher S, King JF (2007). Antiplatelet agents for preventing pre-eclampsia and its complications. *Cochrane Database of Systematic Reviews*.

[13] Duley L, Matar HE, Almerie MQ, Hall DR (2008). Alternative magnesium sulphate regimens for women with pre-eclampsia and eclampsia. *Cochrane Database of Systematic Reviews*.

[14] Hofmeyr GJ, Roodt A, Atallah AN, Duley L (2003). Calcium supplementation to prevent pre-eclampsia—a systematic review. *South African Medical Journal*.

[15] Meher S, Duley L (2006). Progesterone for preventing pre-eclampsia and its complications. *Cochrane Database of Systematic Reviews*.

[16] Meher S, Duley L (2006). Garlic for preventing pre-eclampsia and its complications. *Cochrane Database of Systematic Reviews*.

[17] Meher S, Duley L (2006). Exercise or other physical activity for preventing pre-eclampsia and its complications. *Cochrane Database of Systematic Reviews*.

[18] Meher S, Duley L (2006). Rest during pregnancy for preventing pre-eclampsia and its complications in women with normal blood pressure. *Cochrane Database of Systematic Reviews*.

[19] Aaserud M, Lewin S, Innvaer S (2005). Translating research into policy and practice in developing countries: a case study of magnesium sulphate for pre-eclampsia. *BMC Health Services Research*.

[20] Poel YHM, Swinkels P, De Vries JIP (2009). Psychological treatment of women with psychological complaints after pre-eclampsia. *Journal of Psychosomatic Obstetrics and Gynecology*.

[21] Van Pampus MG, Wolf H, Weijmar Schultz WCM, Neeleman J, Aarnoudse JG (2004). Posttraumatic stress disorder following preeclampsia and HELLP syndrome. *Journal of Psychosomatic Obstetrics and Gynecology*.

[22] Cowan J (2005). *Women's Experience of Severe Early Onset Pre-Eclampsia: A Hermeneutic Analysis*.

[23] Markovic M, Manderson L, Schaper H, Brennecke S (2006). Maternal identity change as a consequence of antenatal hospitalization. *Health Care for Women International*.

[24] http://www.aapec.org.au.

[25] Olde E, Van Der Hart O, Kleber R, Van Son M (2006). Posttraumatic stress following childbirth: a review. *Clinical Psychology Review*.

[26] Nicolaou M, Rosewell R, Marlow N, Glazebrook C (2009). Mothers’ experiences of interacting with their premature infants. *Journal of Reproductive and Infant Psychology*.

[27] Eriksson BS, Pehrsson G (2002). Evaluation of psycho-social support to parents with an infant born preterm. *Journal of Child Health Care*.

[28] McDonald SD, Best C, Lam K (2009). The recurrence risk of severe de novo pre-eclampsia in singleton pregnancies: a population-based cohort. *British Journal of Obstetrics and Gynaecology*.

[29] Hanson S, Hunter LP, Bormann JR, Sobo EJ (2009). Paternal fears of childbirth: a literature review. *Journal of Perinatal Education*.

[30] Keenan A, Joseph L (2010). The needs of family members of severe traumatic brain injured patients during critical and acute care: a qualitative study. *Canadian Journal of Neuroscience Nursing*.

[31] Rothen HU, Stricker KH, Heyland DK (2010). Family satisfaction with critical care: measurements and messages. *Current Opinion in Critical Care*.

[32] Shieh C, Halstead JA (2009). Understanding the impact of health literacy on women’s health. *Journal of Obstetric, Gynecologic, and Neonatal Nursing*.

